# The complete mitochondrial genome of the yellow coloured honeybee *Apis mellifera* (Insecta: Hymenoptera: Apidae) of New Zealand

**DOI:** 10.1080/23802359.2017.1422401

**Published:** 2018-01-03

**Authors:** Ikumi Nakagawa, Mito Maeda, Mao Chikano, Hisashi Okuyama, Robert Murray, Jun-Ichi Takahashi

**Affiliations:** a Faculty of Life sciences, Kyoto Sangyo University, Kyoto, Japan;; b Tai Tokerau Honey Ltd, Kaitaia, New Zealand

**Keywords:** Western honeybee, Illumina sequencing, *Apis mellifera ligustica*, New Zealand, subspecies

## Abstract

The complete mitochondrial genome of the yellow coloured honeybee *Apis mellifera* from North Island, New Zealand was analyzed using next-generation sequencing. The mitochondrial genome was a 16,349bp circular molecule and was predicted to contain 13 protein-coding genes (PCGs), 22 tRNA genes and two rRNA genes. The initiation codon ATA was found in two genes, ATG in four genes, ATT in six genes, and ATC in one gene, while the termination codon TAA was observed in all the PCGs. Phylogenetic analysis using the sequence of 23 closely related taxa suggested a sister relationship with the Italian strain *A. mellifera ligustica*.

The western honeybee, *Apis mellifera* is naturally distributed in African, the Middle East, and Europe, and is exported worldwide. The black honeybee strain *A. m. mellifera* was imported from the UK to New Zealand in 1839 (Beard 2015). Later, New Zealand further imported two subspecies, *A. m. carnica* and *A. m. ligustica*. Honeybees have been imported from abroad until the 1980s, but imports have now been prohibited to control diseases (Beard 2015). The imported honeybees were bred for beekeeping in New Zealand, but some honeybees were naturalized in the bush biome. A record from 1860 reports that New Zealand-made honey was sold by the Maori tribe. Commercial beekeeping in New Zealand began after the Langstroth’s Hive was introduced in 1870 (Beard 2015).

Here, we report the complete mitochondrial genome of the yellow coloured honeybee *A. mellifera* found in New Zealand, which will be useful for phylogenetic studies involving the New Zealand honeybees and other *A. mellifera* subspecies. Adult workers of the yellow coloured *A. mellifera* were collected in March 2017 from Kaitaiya, North Island, New Zealand. Genomic DNA isolated from one worker was sequenced using Illumina’s HiSeq platform. The resultant reads were assembled and annotated using the MITOS web server (Germany; Bernt et al. 2013) and Geneious R9 (Biomatters, Auckland, New Zealand). A phylogenetic tree was constructed using MEGA6 (Tamura et al. 2013) and TREEFINDER (Jobb et al. 2004) using the nucleotide sequences of 13 protein-coding genes (PCGs).

The yellow coloured *A. mellifera* mitochondrial genome was found to form a closed loop that is 16,349 bp long (AP018435). The yellow coloured *A. mellifera* mitochondrial genome represented a typical hymenopteran pattern and was similar to the common *A. mellifera* mitochondrial genome organization, comprising 13 PCGs, 22 putative tRNA genes, two rRNA genes and an A + T-rich control region. The average AT content of the *A. mellifera* mitochondrial genome was 84.91%. Similar to the honeybee mitochondrial genomes, the heavy strand encoded nine protein-coding genes and 14 tRNA genes, and the light strand encoded four protein-coding genes, eight tRNAs, and two rRNA genes. The *ATP6* and *ATP8* genes shared 19 nucleotides. Six protein-coding genes of the *A. mellifera* mitochondrial genome started with *ATT*, the *ATP6*, *COIII, ND4,* and *Cytb* genes started with ATG, the *COI* and *ND3* genes started with ATA, and the *ND2* gene stared with ATC, all of which have been commonly found in the *A. mellifera* subspecies mitochondrial genome (Crozier and Crozier 1993; Gibson and Hunt 2015; Hu et al. 2015; Haddad 2015; Eimanifar et al. 2016a, 2016b; Eimanifar et al. 2017a, 2017b, 2017c; Haddad et al. 2017). The stop codon of each of these protein-coding genes was either TAA, similar to that in the other honeybee subspecies. All the tRNA genes typically possessed cloverleaf secondary structures, except for *Gln*, *Ser1*, and *Thr*, which lacked the arm. Phylogenetic analysis was conducted using the sequence information of 13 mitochondrial PCGs from 23 closely related taxa (Figure 1). The New Zealand yellow coloured *A. mellifera* was most closely related to the Italian subspecies *A. m. ligustica* among all the *A. mellifera* subspecies with sequence homologies ranging from 0.009 to 0.0171.

**Figure 1.**
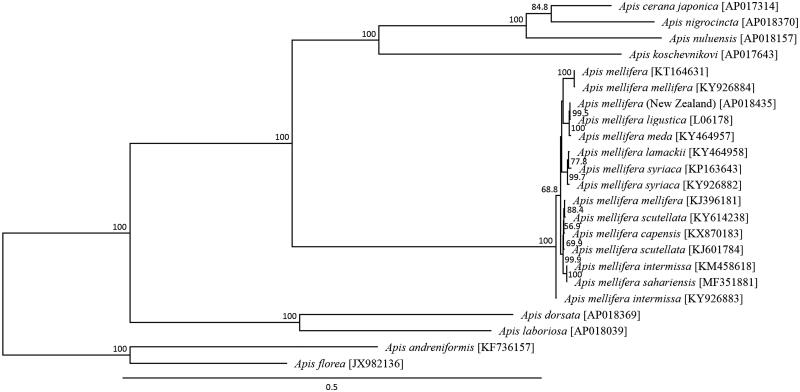
Phylogenetic relationships (maximum likelihood) among the species of the genus *Apis* (Hymenoptera), as determined using the mitochondrial DNA nucleotide sequences of the 13 protein-coding genes. The numbers beside the nodes are the percentages of 1,000 bootstrap values. *Apis florea, A. andreniformis, A. dorsata, A. laboriosa, A. cerana, A. nigrocincta,* and *A. koschevnikovi* (Takahashi et al. 2016; Takahashi et al. 2017a, 2017b, 2017c, 2017d, ; Wakamiya et al. 2017) were used as an outgroup. The alphanumeric terms in the parentheses indicate the GenBank accession numbers.
